# Barriers and alternatives to pediatric rheumatology referrals: survey of general pediatricians in the United States

**DOI:** 10.1186/s12969-015-0028-6

**Published:** 2015-07-29

**Authors:** Colleen K. Correll, Logan G. Spector, Lei Zhang, Bryce A. Binstadt, Richard K. Vehe

**Affiliations:** Division of Rheumatology, Department of Pediatrics, University of Minnesota, East Bldg Rm M668 2450 Riverside Ave, Minneapolis, MN USA; Division of Epidemiology and Clinical Research, Department of Pediatrics, University of Minnesota, Minneapolis, MN USA; University of Minnesota Masonic Cancer Center, Minneapolis, MN USA; Clinical and Translational Sciences Institute, University of Minnesota, Minneapolis, MN USA

**Keywords:** Pediatrician, Pediatric rheumatology, Access to care

## Abstract

**Background:**

Access to pediatric rheumatology (PR) care is limited, however the impact that limited access to PR has on pediatricians has not been examined. The goal of this study was to investigate barriers to PR referrals and resulting alternative referral patterns among primary pediatricians.

**Methods:**

A web-based survey was emailed to primary pediatricians practicing in Minnesota, North Dakota, and South Dakota in order to investigate access to PR care issues. Basic descriptive analysis was performed.

**Results:**

The response rate was 15 % (93/609). Twenty-nine percent (27/92) of respondents’ clinics were at least two hours by car from a pediatric rheumatologist, and 9 % (8/92) were more than six hours away. Ninety-two percent (85/92) had referred a patient to PR at least once, but 89 % (83/93) had experienced a situation in which they considered a referral to PR but ultimately did not. Many had referred to other subspecialists instead: 29 % (24/83) to pediatric infectious disease, 20 % to adult rheumatology, and 12 % to pediatric orthopedics, while 34 % managed the patient themselves. Thirty-five percent (32/60) had referred to an adult rheumatologist, commonly due to decreased travel (44 %), while physician preference was never selected as a reason.

**Conclusion:**

Pediatricians often refer children with possible rheumatic disease to specialists other than PR mainly due to long travel distances. Referral to adult rheumatologists occurs, but not based on pediatrician preference. These findings suggest that the PR workforce is inadequate to meet demand, at least in the Upper Midwest. Interventions are needed to improve access to PR care.

## Background

Rheumatic diseases affect approximately 300,000 children in the United States (US) [1]. Roughly 300 board-certified pediatric rheumatologists in the US care for these children whose diseases are often complex and chronic [2]. As of 2013, 11 states had no pediatric rheumatologist, and an additional 15 states had only one or two residing in the state [3]. Additionally, pediatric rheumatologists are geographically concentrated because most are affiliated with academic institutions [2]. As a result, 40 % of children live more than 40 miles from a pediatric rheumatology (PR) clinic, and 24 % live more than 80 miles from one [4]. Although limited access to PR care is a recognized issue in pediatrics, little is known regarding how this limited access affects management and referral decisions among general pediatricians who encounter children with confirmed or suspected rheumatologic diseases. The goal of this study was to investigate barriers to PR referrals and resulting alternative referral patterns among primary pediatricians.

## Methods

### Physician study population

**Fig. 1:**
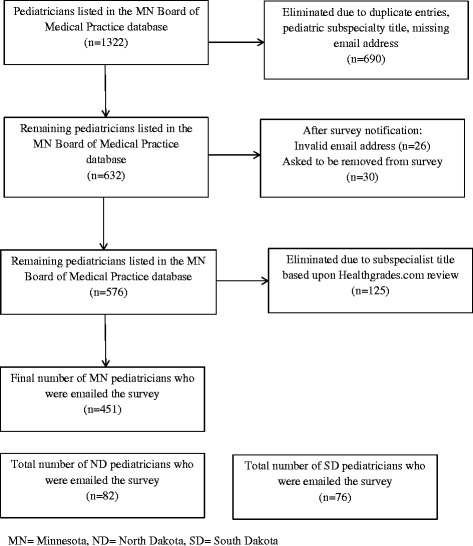
Primary pediatrician study population for MN, ND, and SD

All general pediatricians practicing in Minnesota (MN), North Dakota (ND) and South Dakota (SD) with a valid email address were eligible for the survey. The MN and ND pediatricians’ email addresses were obtained from the MN Board of Medical Practice and the ND Board of Medical Examiners. The SD pediatricians’ email addresses were obtained from the SD chapter of the American Academy of Pediatrics. The MN Board of Medical Practice database contained contact information for 1322 pediatricians. Of these, 690 were eliminated due to duplicate entries, missing email address, or because the physician was not a general pediatrician. This left 632 MN pediatrician email addresses. When a notification about the survey was sent one week prior to the survey, 26 email addresses were found to be invalid and 30 physicians asked to be removed from the survey list. An additional 125 pediatricians were eliminated because they were determined to be pediatric subspecialists based upon a systematic review of each remaining physician in the database using Healthgrades.com. Thus, the survey was emailed to a total of 451 MN pediatricians (Fig. 1). The survey was also emailed to a total of 76 pediatricians practicing in SD and 82 practicing in ND, however, the investigators did not have direct access to these databases; rather the link to the survey was forwarded to the pediatricians by the administrators of the databases. This survey was determined to be exempt from formal institutional review board (IRB) review at the University of Minnesota.

### Instrument

The survey queried primary pediatricians to assess their experience with pediatric rheumatic diseases, perceived barriers to access to PR care and reasons for referring patients to a PR clinic. In order to assess the pediatricians’ experience with pediatric rheumatology, the survey questions included assessments such as whether or not the respondent’s residency training institution had a section of PR, the types of rheumatic diseases encountered and diagnosed both during and after pediatric residency training, and whether continuing medical education (CME) credits were focused on pediatric rheumatology. Regarding the barriers to access to care, pediatricians were asked how far their clinic was from a PR clinic, whether they had ever referred a patient to a PR clinic, reasons for referring to PR, reasons why they did not refer to a PR clinic when they considered it, and whether they had ever referred to an adult rheumatologist and the reasons for doing so. The survey consisted of multiple choice answers and “select all that apply” answers with options to add free text.

### Survey administration

The survey was conducted between June 19, 2013 and July, 10 2013 using the web-based program, Research Electronic Data Capture (REDCap™). One week prior to sending the survey, a notification email was sent. A link to the survey was sent to the pediatricians on June 19, 2013. The survey was open for three weeks. A reminder email was sent one week prior to the closing of the survey. As an incentive to complete the survey, those who completed the survey were entered into a drawing to win a $400 gift card for an online retailer.

### Analysis of responses

Responder and non-responder characteristics were compared using two-sample t-tests for continuous variables and Fisher’s exact test for categorical variables. General frequency distributions of responses were also performed.

## Results

The overall response rate was 15 % (93/609), and the response rates from each state were 16 % (70/451) for MN, 13 % (10/76) for ND, and 13 % (11/82) for SD. Comparisons between respondents and non-respondents were performed only for the MN pediatricians because the investigators did not have access to the ND or SD databases. Responders and non-responders were compared by gender, age, years of practice and geographic setting of their clinic (urban, suburban, rural). Responders tended to be younger (mean age 48 yo versus 54 yo, p = 0.0005), were more likely to be female (69 % vs. 49 %) and had been in practice for a shorter period of time (mean 22 y versus 27 y, p = 0.0051). However further analysis determined that among both responder and non-responder groups, women tended to be younger. Therefore the gender difference between the responders and non-responders is merely reflective of the age difference. There was no significant difference between responders and non-responders regarding geographical setting of the clinic. The demographics of the respondents are shown in Table 1.

**Table 1:** Demographic comparison among responders and non-responders using MN data only

Respondent Demographics	Responders	Non-Responders	*P*-value
N (%)	93 (15)	516 (85)	
Mean age in years (range)	48 (33-70)	54 (31-84)	0.0005
Mean years in practice (range)	22 (6-45)	27 (2-58)	0.0051
Self-reported geographic location of clinic			0.2390
Urban: N (%)	37 (40)	254 (49)	
Suburban: N (%)	37 (40)	209 (41)	
Rural: N (%)	18 (20)	53 (10)	
Male	29 (31)	262 (51)	0.0042

### Referral patterns to PR

Ninety-two percent (85/92) of surveyed pediatricians had referred a patient to a pediatric rheumatologist. The most commonly selected reasons for referral to PR were for high suspicion for a rheumatic disease (88 %), chronic arthritis of unclear etiology (64 %), musculoskeletal (MSK) pain with an otherwise normal exam (44 %) and positive antinuclear antibody (ANA) of unclear significance (44 %).

### Barriers to rheumatology referrals

Sixty-four percent (59/92) of respondents reported that their clinic was within a one hour drive from the nearest PR clinic, however 29 % reported that their clinic was more than two hours away and 9 % reported being more than six hours away. Eighty-nine percent (83/93) of respondents had encountered a situation in which they considered referring a patient to a pediatric rheumatologist but ultimately did not. The three most commonly selected reasons for not referring to a PR clinic in this circumstance were that the pediatrician managed the patient independently (34 %), the patient’s condition improved while waiting to be seen by a pediatric rheumatologist (29 %) and the patient was referred to a pediatric infectious disease specialist instead (28 %). Twelve percent referred to a pediatric orthopedist and 4 % referred to a general orthopedist (Table 2).

**Table 2:** Reasons that potential referrals to a pediatric rheumatologist were not completed

Response*	Percent (n = 83)
I managed the patient myself	34
The patient improved while waiting to be seen	29
I referred to a pediatric infectious disease specialist instead	28
I referred to adult rheumatologist instead	20
I referred to a pediatric orthopedist instead	12
I referred to a pediatric hematology/oncology specialist instead	9
Other	9
The distance or time to travel was too long	8
I referred to a pediatric sports medicine specialist instead	4
I referred to a general orthopedist instead	4
The patient’s insurance would not cover a PR referral	1

*Respondents were asked: In cases in which you considered a pediatric rheumatology referral but did not refer a patient to a pediatric rheumatologist, please indicate your reasons for not doing so. (Select all that apply)

When asked if they had ever referred a pediatric patient to an adult rheumatologist, 35 % (32/92) reported that they had. Among the listed reasons for doing so, the most common responses included that the patient was an adolescent and could be managed by an adult rheumatologist (47 %) and shorter travel time or distance to the adult rheumatologist (44 %). An additional 9 % added long wait-time to see a pediatric rheumatologist as a reason for referring to an adult rheumatologist despite wait-time not being a pre-specified choice in the survey. Insurance reasons and family preference were rarely selected, and none of the respondents selected physician preference as a reason for referring a pediatric patient to an adult rheumatologist.

### Experience with PR

**Fig. 2:**
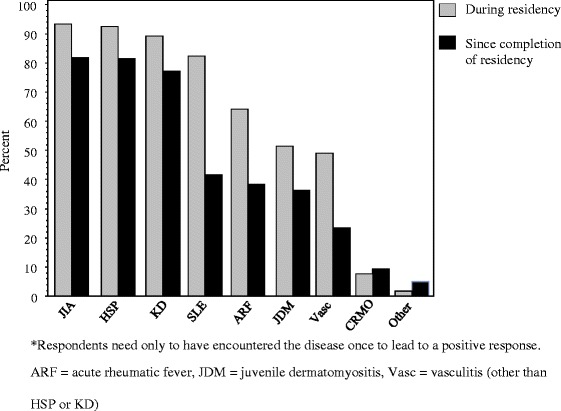
Diseases encountered during and since completion of pediatrics residency

Eighty-five percent (79/93) of respondents had spent less than four weeks and 48 % had spent no time in a PR clinic during their residency training. The respondents were asked about their encounters with a variety of pediatric rheumatic conditions, and at least 80 % of respondents had encountered juvenile idiopathic arthritis (JIA), Henoch-Schonlein purpura (HSP), Kawasaki disease (KD), and systemic lupus erythematosus (SLE) during residency. There was a significant decrease in the percentage who had encountered a patient with SLE since completion of residency (42 %). Only 52 % of respondents had been exposed to juvenile dermatomyositis (JDM) and 50 % had been exposed to vasculitis during residency. In general, residency training appears to have provided a greater breadth of exposure to PR diseases than practice apart from chronic recurrent multifocal osteomyelitis (CRMO) and “other” diseases (Fig. 2).

Thirty-one percent (29/93) stated that over the last three years, a component of continuing medical education (CME) was focused on PR.

## Discussion

The vast majority of primary pediatricians responding to this survey had referred a patient to a PR clinic. The most common reasons for doing so were because they had been concerned about a patient having chronic arthritis or other rheumatic disease, for non-specific MSK pain or for a positive ANA test. These findings parallel those of a previous study which demonstrated that the most common referrals to a pediatric rheumatology center were for MSK pain, abnormal lab tests, and joint swelling [5]. However, the majority of respondents in our study have also been in a situation in which they considered referring a patient to a PR clinic but ultimately did not. In these circumstances, the most common reasons given for not referring the patient were that the pediatrician managed the patient themselves or that the patient improved while waiting to be seen. That they managed the patient themselves is notable, and somewhat concerning because a previous study pointed out that many pediatricians do not feel comfortable managing rheumatologic diseases [6]. The current study also revealed that most of the pediatricians have had limited duration of exposure to pediatric rheumatologic diseases during training. Therefore it is probable that in cases in which the pediatrician managed the patient themselves, the patients did not have a chronic rheumatic disease. However, if general pediatricians are managing chronic rheumatic diseases without a subspecialist’s guidance, it would be important to identify the types of diseases they are treating and how their management compares to that of a pediatric rheumatologist. This survey was not designed to address these points.

Interestingly, in cases in which a pediatrician considered a PR referral but ultimately did not complete the referral, the most common alternative subspecialty referral was to a pediatric infectious disease specialist and less commonly to pediatric or general orthopedics. In comparison, a previous study demonstrated that the majority of children with oligoarticular JIA were referred by their primary care physician to an orthopedic surgeon before being referred to a pediatric rheumatology clinic [7]. Therefore it would be important to know whether the children who were referred to an infectious disease specialist had unique features such as recurrent or persistent fever, lacked MSK involvement, or had features of diseases commonly treated by infectious disease physicians including KD, acute rheumatic fever, or Lyme arthritis.

Not surprisingly, one-third of surveyed pediatricians had referred a pediatric patient to an adult rheumatologist, and the most common reason for doing so was that the patient was an adolescent. It has previously been reported that more than 60 % of adult rheumatologists had cared for pediatric patients [8]. However the majority limited the types of diseases they would treat to JIA and SLE and would only treat children who were at least 6 years old [9]. Although some diseases such as SLE and rheumatoid factor-positive polyarticular JIA affect children and adults similarly, many of the pediatric rheumatic diseases occur essentially only in childhood, and therefore patients with these diseases are likely better served by a pediatric rheumatologist.

According to this survey, it appears that from the primary pediatrician’s perspective, wait-time is an equally important barrier to PR care as distance. This finding is consistent with a previous survey of general pediatricians in which 62 % indicated wait-time as a moderate to significant barrier to access to subspeciality care compared to 37 % who indicated travel distance as a barrier [10]. Although 29 % of respondents reported that their clinic was more than a two-hour drive from the nearest PR clinic, distance was the reason for not referring to a PR clinic only 9 % of the time. Moreover, it is likely that an even higher percentage of respondents would have selected wait-time as a reason if it had been listed as a response option. This is a particularly revealing observation especially in this geographic region of the Upper Midwest where distances to travel are great. This illustrates that, at least from the primary care provider’s perspective, the key to improving access to PR care might best be addressed by increasing the number of pediatric rheumatologists rather than increasing the geographic distribution of pediatric rheumatologists. However, distance to travel may be a greater issue for patients and families than is realized by their primary care providers.

Two important limitations to this study are the low response rate and the inclusion of participants only from three states of the Upper Midwest. Due to the low response rate, the results of the survey may not be generalizable to all pediatricians and this makes interpretation of results difficult. The response rate was low despite attempts to improve responses by sending an introductory email, sending a reminder email, and providing a gift incentive. However, low response rates are common for physician surveys [11]. The only statistically significant differences between respondents and non-respondents were age, years in practice, and gender. Age and years in practice would be expected to be linked. Further analysis determined that the gender difference was likely reflective of age, with women physicians tending to be younger in this survey. The higher response rate from younger individuals presumably reflects their tendency to attend more routinely to their email accounts compared to older individuals. Despite this difference in age, the characteristics of the respondents were representative of the primary pediatric workforce in MN [12].

The survey was limited to MN, ND, and SD because these states are a part of the catchment area for the investigators’ PR clinic. The Upper Midwest has more rural populations with greater travel distances to pediatric subspecialists which contrasts from highly populated regions of the US. Moreover, it is possible that the majority of respondents trained in the small number of pediatric residency programs in the region, leading to relative homogeneity of their training experiences. Therefore the findings from this survey may not be representative of all primary pediatricians, particularly those practicing in dissimilar geographic and demographic regions of the United States.

Another limitation of the study was that it focused solely on general pediatricians and did not include family physicians. It would be important to capture viewpoints from family physicians as they represent 50 % of the primary care workforce in MN [12]. Moreover national data show that approximately 20 % of child health care is provided by family practioners, and family physicians in rural areas are more likely to provide health care to children. [13–15]. An important future study would include family practitioners and compare their responses to general pediatricians’ responses.

## Conclusion

This study illustrates that general pediatricians face challenges to PR referral due to long wait-time and distance to travel and thus often choose alternative measures including caring for the patients independently and/or referring to other specialists who may or may not have appropriate training to manage these diseases. These results strongly suggest significant opportunity and need to increase access to PR care for children with suspected rheumatic diseases. Increasing access is not a simple task. Findings from this survey indicate that increasing the number of practicing pediatric rheumatologists in order to reduce wait-time is at least as important as decreasing the geographic distance to a PR clinic. Additional interventions that might be beneficial include establishment of outreach clinics or use of telemedicine, but to implement and evaluate the impact of such interventions again highlights a need an increased number of practicing pediatric rheumatologists. For example, to address the issue of limited access in Alaska, Seattle Children’s Hospital has established an outreach clinic in Alaska, however to our knowledge outcome measures have not been studied and published [16]. Telemedicine, which has been proven successful in adult rheumatology, is one of the proposed policy changes to address workforce issues in pediatric rheumatology, both nationally and internationally [17–20], but whether this would satisfy the desires of referring physicians is not yet known. This survey did not directly assess pediatricians’ desire for either of these interventions, but these questions will be evaluated in upcoming studies. Likewise, this survey did not address pediatricians’ satisfaction with the level of service provided by existing regional PR clinics, but would be a helpful part of comprehensive assessment of service needs and service planning. Finally, this survey was limited to a small portion of primary pediatricians practicing in the Upper Midwest of the US, and the generalizability of these findings requires confirmation by further studies that include larger numbers of practicing pediatricians and family practitioners from this and other geographic regions of the US.
